# No relation between fMRI findings and thought distortion in mood disorder? A claim for new studies

**DOI:** 10.3389/fpsyt.2025.1435227

**Published:** 2025-03-07

**Authors:** Adriana Munhoz Carneiro, Pedro Henrique Rodrigues da Silva, Valquiria Aparecida da Silva, Andre Russowsky Brunoni

**Affiliations:** ^1^ Service of Interdisciplinary Neuromodulation, Department and Institute of Psychiatry, University of São Paulo Medical School, São Paulo, Brazil; ^2^ Mood Disorders Unit, Faculty of Medicine FMUSP, University of São Paulo, São Paulo, SP, Brazil

**Keywords:** depression, thoughts, cognitive distortion, fMRI, thinking, review

Depression and bipolar disorder are two of the most common disability diseases worldwide (1). Despite extensive research, treatment remains challenging: around 30%–50% of patients do not respond to initial pharmacological treatments, and only 40% achieve complete symptom remission (2). From the non-pharmacological alternatives, electroconvulsive therapy (ECT) can achieve higher response rates; however, the biological mechanisms related to this response are still unclear.

Considering the complexity of depression and bipolar disorders, treatment requires a higher level of specificity to achieve better response and adherence, as well as to lower their incidence. Recent efforts to identify functional neural substrates related to depressive symptoms are a promising direction to achieve this goal. Network theories of depression (3–5) suggest that depression arises from complex interactions among symptoms rather than being a distinct disorder, which complicates the search for specific neural correlates of depression. However, a recent study has already shown positive results of mapping symptom-specific profiles on the improvement of depressive symptom treatment response (6).

Directly related to the treatment options, we note that thought distortions are considered one of the most common symptoms of depressive episodes. Several models have been proposed for its understanding, with the cognitive triad model (7) being one of the most likely to endorse its complexity, defining distorted thoughts as depressive symptoms. According to this model, patients with depressive episodes might present thought distortions in the form of a negative view of themselves, of others, and/or the future (8). These might manifest in the form of negative assumptions or automatic errors, causing patients to become catastrophic or ruminative: the more distortion, the more severe the symptoms.

However, previous studies have shown that patients might recover from depressive symptoms, but still with their thought distortions, in both pharmacological (9) and non-pharmacological treatments (10), which may be interpreted in two ways: 1) a trait, meaning a relatively enduring characteristic influenced by genetics and the environment, similar to personality, or 2) a state, i.e., considering those distortions as temporary conditions, linked to depressive episodes, that tend to decrease as patients recover from the disease (8, 11). Understanding the relationship between thought contents, functional magnetic resonance imaging (fMRI) findings, and mood disorders might be valuable for a better integrated practice. By investigating the brain function through fMRI, we may be able to investigate better approaches to understanding the mechanisms of action that are correlated with higher rates of treatment response.

We decided to perform a systematic review, the aim of which was to understand how cognitive distortions can be related to fMRI findings. We considered that multimodal combinations of neuroimaging with clinical findings—collected through scales and interviews— would contribute to a better understanding of the impact of neural networks and, consequently, to a better treatment approach. For this search, we considered four electronic databases (Scopus, Embase, MEDLINE through PubMed, and Web of Science) with no restriction of time or language, up to May 2024. The keywords used were as follows: (depression; major depression; MDD) (for depression); bipolar, bipolar disorder, BD (for bipolar disorder); thought distortions, cognitive distortion, cognitive errors (for distortions); and fMRI, functional magnetic resonance imaging (for neuroimage). No results were found from this search.

The lack of studies in this area shows the need for more integrative solutions for patients suffering from depression and bipolar disorder. The use of fMRI findings might contribute to identifying possible predictors of response and possible mechanisms of action in patients undergoing neurostimulation techniques (12). In the same sense, identifying specific clusters of patients with thought distortions typically related to depressive episodes, i.e., “the higher ruminative” or “the higher catastrophizing” might also contribute to more effective and more personalized pharmacological and psychosocial treatments. By neglecting the impact of one of the most studied depressive symptoms, we might be missing important information about how those thoughts are modulated and the main impact of a more precise therapy.

Finally, understanding the interplay between brain networks and thought distortions has the potential to improve the therapeutic interventions and outcomes for individuals with depression. Future research addressing these unanswered questions can pave the way for more targeted and effective treatment approaches. We recommend that future studies incorporate comprehensive data collection, focusing on the duration of disease, the use of medication, the frequency of episodes, and the use of standardized measures to assess thinking distortions. These insights will strengthen the methodological rigor of research and may reveal key factors that influence treatment effectiveness, as well as the development of more individualized and personalized treatment approaches.
